# An Intersectional‐Contextual Approach to Racial Trauma Exposure Risk and Coping Among Black Youth

**DOI:** 10.1111/jora.12757

**Published:** 2022-04-19

**Authors:** Chardée A. Galán, Evan E. Auguste, Naila A. Smith, Jocelyn I. Meza

**Affiliations:** ^1^ University of Southern California University of Southern California Los Angeles United States; ^2^ 5923 Fordham University; ^3^ Dickinson College; ^4^ The Pennsylvania State University; ^5^ University of California, Los Angeles

**Keywords:** adverse childhood experiences, racial trauma, racism, intersectionality, gendered racism

## Abstract

Black youth experience racial discrimination at higher rates than other racial/ethnic groups in the United States. To identify how racism can simultaneously serve as a risk factor for adverse childhood experience (ACE) exposure, a discrete type of ACE, and a post‐ACE mental health risk factor among Black youth, Bernard and colleagues (2021) proposed the culturally informed ACEs (C‐ACE) model. While an important addition to the literature, the C‐ACE model is framed around a single axis of race‐based oppression. This paper extends the model by incorporating an intersectional and ecodevelopmental lens that elucidates how gendered racism framed by historical trauma, as well as gender‐based socialization experiences, may have implications for negative mental health outcomes among Black youth. Clinical and research implications are discussed.

The frequency, nature, and impact of racism on Black youth likely differ across genders and contexts (Assari et al., 2017; Hughes, Watford, et al., 2016), underscoring the need for an intersectional‐contextual framework to understanding racism and trauma. An intersectional‐contextual lens recognizes that racism (unequal system of power that privileges white people and oppresses Black people) interacts with other types of oppression like sexism (social system that creates gender inequality; Collins, 2000). This interaction gives rise to unique manifestations of racism along gendered lines (Collins, 2000; Crenshaw, 1991) that also varies within and across contexts (Hughes, Watford, et al., 2016; Saleem et al., 2020). Theoretical models of adverse childhood experiences (ACEs), which are negative life events that put children at risk for a range of poor life outcomes including trauma (Herzog & Schmahl, 2018), have largely neglected stressors uniquely affecting Black youth, such as racism, and often fail to consider the role of context across development. Recent theories such as the culturally informed ACEs model (C‐ACEs) have sought to address this gap by incorporating a focus on racism into thinking about ACEs (Bernard et al., 2021). The C‐ACE model extends our understanding of the traumatic effects of racism on Black youths’ mental health by identifying racism as an ACE, as well as a factor that puts Black youth at risk for other ACEs, and poor post‐ACE outcomes (Bernard et al., 2021). While the C‐ACE model is decontextualized, the Developmental and Ecological Model of Youth Racial Trauma (DEMYth‐RT) explicitly focuses on how ecological contexts are both sources of risk for and coping with ACEs (Saleem et al., 2020), the latter of which is rare among trauma‐focused theoretical models. Nevertheless, a major limitation of both models is a focus on a single axis of oppression (i.e., racism), neglecting other systems of oppression affecting Black youth, such as sexism.

Much of the literature on Black youths experiences of racism has focused on interpersonal experiences of differential treatment by race (i.e., racial discrimination, microaggressions). This research shows that Black youth experience racial discrimination at significantly higher rates than any other ethnic‐racial minority group in the United States (U.S.) (Pachter & Coll, 2009), averaging five discrimination experiences per day (English et al., 2020). The frequency of racial discrimination exposure can vary across contexts (e.g., school, neighborhoods), time, and perpetrator (teachers vs. peers; Greene et al., 2006; Hughes, Del Toro, et al., 2016), and these experiences are associated with poorer mental health including elevated levels of depression, anxiety, and suicidality (Benner et al., 2018; Galán et al., 2021; Loyd et al., 2019). More recently, scholars have suggested that the enduring, pervasive effects of racial discrimination can lead to trauma symptoms (e.g., intrusive thoughts, hypervigilance; Carter, 2007; Heard‐Garris et al., 2018), coining the term “racial trauma” (Comas‐Díaz et al., 2019). A growing body of research primarily among Black adults shows that racism is gendered (e.g., Williams & Lewis, 2019). Gendered racism describes unique forms of racism by gender and heightened vulnerability across contexts (e.g., Black girls and sexual violence; Essed, 1991). To truly examine and respond to the gendered and contextual nature of mental health vulnerabilities among Black adolescents, research needs to examine the historical and contemporary experiences of racism that shape Black adolescents’ mental health across different contexts. This article takes an intersectional‐contextual perspective on racism by drawing on classic theories and contemporary trauma models to review the literature on gendered racism during key developmental contexts for Black adolescents. Taking a strengths‐based approach, we also focus on cultural coping mechanisms that may facilitate better mental health outcomes for Black adolescent boys and girls.

## THEORETICAL FRAMEWORK

In this review, we draw from contemporary trauma‐focused theories (C‐ACEs, DEMYth‐RT) and classic frameworks (intersectionality, ecodevelopmental) to advance an intersectional‐contextual approach to understanding the gendered nature of racism as an adverse childhood event across contexts. First, we draw on the C‐ACE model, a trauma‐focused model that extends the ACE framework by positing that racism (1) increases Black youths’ exposure to potentially traumatic events (e.g., involvement with juvenile justice system); (2) is a distinct ACE category through cultural (e.g., dehumanizing stereotypes), interpersonal (e.g., racial discrimination), and institutional (e.g., disproportionality in school discipline outcomes) mechanisms, and; (3) influences post‐ACE mental health outcomes for Black youth (Bernard et al., 2021). In this review, we focus on the parts of the model that link historical trauma to contemporary racist social conditions, laying the groundwork for racism as a distinct ACE for Black youth. We also draw on intersectionality, which suggests that economic, political, and ideological oppression vary along racial and gendered lines to suggest that racism as an ACE may vary by gender (Crenshaw, 1991). Accordingly, Black girls and boys likely have distinct experiences of racism because of different gendered racial stereotypes (e.g., Black boys stereotyped as dangerous, violent, criminal; Black girls stereotyped as sexually promiscuous, lascivious, and servile; Collins, 2000).

**Figure 1:**
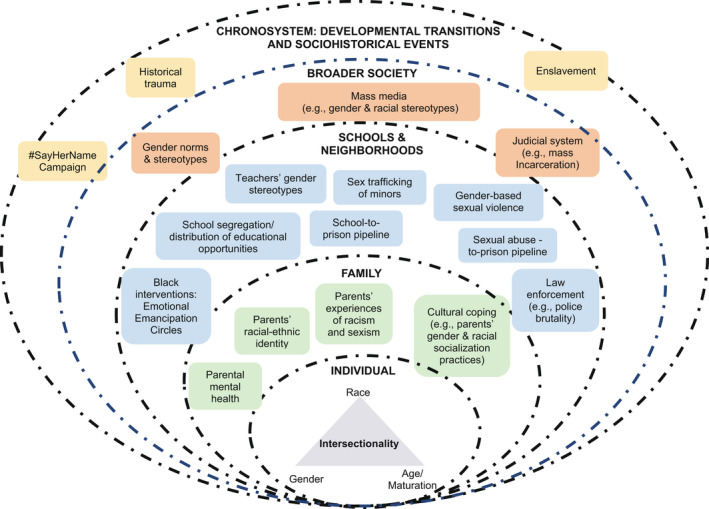
An intersectional‐contextual approach to racial trauma exposure risk and coping among Black youth.

To this intersectional lens, we apply a contextual approach, which is common in the trauma literature. For instance, DEMYth‐RT posits that chronic exposure to racism (e.g., institutional, interpersonal) can put youth at risk for racial trauma but social and cultural assets can facilitate coping with racial trauma (Saleem et al., 2020). Here, we draw specifically on the ecodevelopmental framework, which blends a focus on context with a focus on risk and protective factors embedded in youths’ environments that shape their mental health outcomes over time (Szapocznik & Coatsworth, 1999). This framework draws on Bronfenbrenner’s ecological model to propose that youth are embedded in multiple, interacting environments, such as the macrosystem (context comprising of cultural ideologies) and microsystem (direct interactions with youth in home, school, and neighborhood contexts; Bronfenbrenner & Morris, 2007; Szapocznik & Coatsworth, 1999). Accordingly, gendered racial stereotypes are part of the macrosystem context that may drive institutional and interpersonal racism (risk factors) over time and in contexts like schools, the juvenile justice system, and neighborhoods (see Figure 1). In this article, we focus on these key contexts because of their increasing salience in adolescence. As youth get older, the stakes of poor school outcomes become higher, they are viewed and treated more like adults, and they spend more unmonitored time in their neighborhoods (Bucci et al., 2021; Ingoldsby & Shaw, 2002), which heightens their vulnerability to risks such as police violence and sex trafficking.

### Cultural Coping: Gendered Racial Socialization

Racism may also spur Black families to engage in cultural coping strategies such as racial socialization in the family microsystem context, which is protective against the negative effects of racism (Anderson & Stevenson, 2019; Hughes et al., 2006; Umaña‐Taylor & Hill, 2020). Parent racial socialization is a multifaceted parenting practice that involves instilling racial and cultural pride in youth and preparing youth for potential racial bias (Hughes et al., 2006). There is evidence that parents engage in gendered racial socialization in recognition of the unique vulnerabilities faced by Black boys and girls (e.g., Peck et al., 2014). Critically, there is evidence that racial socialization can buffer the detrimental effects of racial discrimination on youth psychological adjustment (Hughes et al., 2006; Wang et al., 2020). As such, we focus on parents’ use of gendered racial socialization to help Black boys and girls cope with gendered racial discrimination in school, justice, and neighborhood contexts.

## CHRONOSYSTEM: HISTORICAL TRAUMA ALONG GENDERED LINES

Historical trauma refers to the idea that collective tragedies throughout history can imprint themselves into the cultural memory of a particular group (Brave Heart & DeBruyn, 1998), and these historical experiences of trauma likely manifested differently for Black men and women in the U.S. (e.g., Brave Heart, 1999). It is important to understand the impact of historical trauma because it has deleterious effects on Black people’s psychological health through myriad pathways (Brave Heart & DeBruyn, 1998; Gone, 2013; Gump, 2010). For instance, when historical injustices (e.g., enslavement; part of the chronosystem context) are reflected upon, these reflections can increase the risk for mental health problems long after those events have passed and among people that never experienced the events themselves (Brave Heart & DeBruyn, 1998). Moreover, historical injustices can affect the contemporary experiences of Black people through the intergenerational transmission of stress and systemic contexts that continue to oppress them (Gone, 2013; Gump, 2010). Society must consider the complexities of historical trauma in order to redress them and ensure their effects do not persist in future generations (National Coalition of Blacks for Reparations in America, 2021).

The transatlantic slave trade is a historical trauma that was undergirded by scientific racism and has informed how Black boys and girls are treated in the U.S. Scientific racism and racial capitalism dehumanized people from Africa, racializing them as Black and intellectually inferior, devaluing and erasing their culture, and treating them as objects to create capital, and as capital themselves (Ani, 1981; da Silva, 2020; Nobles, 1978; Spillers, 1987; Smedley & Smedley, 2005; Wilderson, 2009). Racist experiences during enslavement varied by gender. For Black women and girls, sexual and reproductive acts of violence were a common occurrence during enslavement (Johnson, 2020). For instance, Black women and girls were routinely subject to nude physical examinations during public auctions to assess their reproductive ability and those who were considered “strong” were then raped to birth more children into slavery for economic profit (Bridgewater, 2005). Young black girls were also encouraged to have sex as preparation for their later status as breeders (Bridgewater, 2005). The erasure of Black girlhood and equating Black women’s worth with their sexual and reproductive abilities shaped the opportunities and treatment of Black women and girls whether enslaved or “free” within the U.S.’s slave‐based economy (Johnson, 2020).

During slavery, Black men and boys experienced different types of violence because they were characterized as prone to physical and sexual violence and therefore needed to be enslaved to protect white people (Kendi, 2019). These stereotypes meant that Black men were subjected to severe physical punishments, which were often publicized and treated as a spectacle (Wilkerson, 2011). For instance, throughout enslavement and the Jim Crow Era, Black men were frequently lynched publicly or executed by the state for unsubstantiated accusations ranging from sexual assault to theft (Wilkerson, 2011). For example, Emmett Till, a 14‐year‐old boy, was brutally murdered in Mississippi in 1955, because he was accused of flirting with a white woman (Library of Congress, n.d.). Despite the extreme physical violence experienced by Black men, the U.S. engaged in targeted recruitment of Black men and boys to fight in every major American war (Alt et al., 2002), leveraging racist stereotypes about their physical prowess (Alt et al., 2002). Even while serving their country, Black soldiers were vulnerable to violence. During the Fort Pillow massacre, confederate soldiers slaughtered ~300 predominantly Black soldiers who had surrendered (Cimprich, 2005). Moreover, recognition for their service and provision of postwar benefits were not equally distributed for Black veterans (Alt et al., 2002), further devaluing their lives and contributions. In sum, while racism during slavery dehumanized and devalued all Black people, historical trauma was gendered: Black girls were at heightened risk of sexual and reproductive violence and Black boys were at heightened risk for physical violence. It is important to note that the recognition of heightened likelihood does not erase the very real and consistent threats of all types of violence for Black boys and girls.

## CONTEMPORARY RACISM: AN INTERSECTIONAL‐CONTEXTUAL APPROACH

Historical trauma due to slavery has been carried forward in contemporary American society through ongoing racist stereotypes that undergird public policies, institutional practices, and interpersonal interactions, which manifest as gendered racism within context. However, Black parents often engage in gendered racial socialization to protect their Black boys and girls from the systemic and interpersonal experiences of racism fueled by harmful stereotypes.

### Broader Society: Gendered Stereotypes of Black Girls and Boys

The stereotypes that undergirded much of the physical and sexual violence experienced by Black people during and immediately after slavery continue to operate in contemporary times (see Figure 1). Notably, the history of sexual exploitation and violence that Black women endured manifested in the Jezebel stereotype, which represents Black women and girls as inherently sexually promiscuous (Collier et al., 2017; Pilgrim, 2002). In the decades following the end of slavery, the Jezebel stereotype continued to put Black women and girls at heightened risk of being beaten and incarcerated for prostitution with no evidence and solely at the discretion of white male police officers (Hartman, 2019). These stereotypes have also led to Black girls being viewed as less innocent and more like adults compared to white girls in contemporary times and vulnerable to disproportionate rates of discipline in schools (Epstein et al., 2017; Morris, 2016).

Similarly, many scholars argue that stereotypes of Black men as violent and dangerous continue to shape how Black men and boys are treated today (Atkins‐Loria et al., 2015; Degruy‐Leary, 1984). Historically, social scientists explained Black boys’ social difficulties as due to their biology or culture (Cross, 2003), fueling stereotypes of Black boys behaviors reflecting violence and criminality (Welch, 2007). For instance, in the 1990s, the “super predator” stereotype reintroduced the myth of irredeemable and predestined Black male criminality that destroyed communities (Moriearty & Carson, 2012). This myth triggered a wave of juvenile justice reforms that resulted in the disproportionately harsh sentencing of Black boys (National Campaign to Reform State Juvenile Justice Systems, 2013). Recent research suggests that these ideas continue to prevail as Black boys are consistently seen as older, less innocent, and more dangerous than same aged, other raced peers (Goff et al., 2014). To conclude, racist gendered stereotypes in contemporary times have contributed to the erasure of Black girlhood and Black boyhood, which has directly shaped institutional policies and practices and their treatment in the school and justice systems.

### Schools and Neighborhoods

#### The school‐to‐prison pipeline

One of the most harmful effects of contemporary gendered racist cultural stereotypes in the lives of Black youth has been its legacy in the education system through the school‐to‐prison pipeline. The school‐to‐prison pipeline describes the unintended consequences of zero tolerance school policies intended to reduce school violence by requiring schools to respond to even minor student disruptions with harsh punishments but often act as a direct pathway to the juvenile justice system (Cerrone, 1999; Christle et al., 2005; Curtis, 2014; Khalek, 2011). Because of these policies, Black boys and girls have been disproportionately disciplined in school contexts beginning as early as preschool. According to the U.S. Department of Education (2018), even though Black boys make up 7.9% of the pre‐K‐12 student population, they account for 25% of expulsions, 33.9% of school‐related arrests, and 29.4% of referrals to law enforcement under zero tolerance policies. In pre‐K‐12 schools, Black girls are the only group of girls subjected to disproportional exclusionary school discipline, at two times the rate of their current enrollment size (U.S. Department of Education, 2018). Black girls are also being channeled into the juvenile justice system via punitive, zero‐tolerance policies but their unique experiences and needs have been rendered largely invisible in this line of work (Crenshaw et al., 2015; Morris, 2016). As such, the school‐to‐prison pipeline not only affects Black boys but also harms Black girls.

Despite commonalities in the disproportionality of school discipline for Black girls and Black boys, there are unique gendered ways in which the school‐to‐prison pipeline manifests. For one, gendered racial stereotypes can lead to microaggressions, which are subtle, often unintentional verbal slights that invalidate or insult the individual because of their race and gender (Ghavami & Peplau, 2013; Sue et al., 2008). Black boys typically experience gendered racial microaggressions that are related to assumptions of inferior intellect and criminality (Sue et al., 2008). These experiences can be frustrating and enraging, leading to increases in aggressive attitudes in Black boys (Cunningham et al., 2018). Displays of aggression are rooted in gendered norms of emotional expression (Berke & Zeichner, 2016), and when aggression is displayed by Black boys, this likely reinforces stereotypes about their behavior and heightens their vulnerability to justice‐contact and being pushed out of the education system. These interpersonal and systemic contexts likely explain why 28% of Black men report never progressing further in their education than a high school diploma compared to 17% of Black women (Black Future Lab, 2019). In essence, the gendered racism and historical trauma of controlling Black boys has reproduced by effectively allowing disproportionately white educators to guide Black boys into the justice system (e.g., Grissom et al., 2009; Lindsay & Hart, 2017; Meier & Stewart, 1992).

A similar gendered‐racial process occurs for girls but is rooted in other kinds of gendered expectations for Black girls’ behavior. Recent research shows that teachers’ subjective evaluations of Black girls behaviors (e.g., compliance) are typically a precipitating factor for referral for exclusionary discipline (Gibson et al., 2019; Girvan et al., 2017). There is evidence to suggest that these discipline referrals tend to enforce white, mainstream standards of femininity, such as being compliant, passive, and quiet (Blake et al., 2011; Crenshaw et al., 2015; Morris, 2007). In one qualitative study, teachers linked loud and insolent behavior to Black girls, chastised them for being “unladylike,” and “the presumed loud and confrontational behavior of African American girls was viewed as a defect that compromised their very femininity” (Morris, 2007, p. 506). Similar stories of racialized gender policing abound in the media, with several stories of Black girls being disproportionately targeted for racist and sexist dress codes, including school policies restricting the use of hair wraps or wearing their natural hair (Dvorak, 2018; Hobdy, 2020; National Women’s Law Center, 2018).

##### Gendered racial socialization to cope in schools

Black boys and Blacks girls receive gendered racial socialization messages from parents aimed at preparing them to deal with negative stereotypes and microaggressions in schools and facilitate adaptive development. Due to structural racism, which has limited Black families access to economic and social resources, Black families have had to rely on Black women’s financial contribution for economic survival (Anderson & Shapiro, 1996; Boustan & Collins, 2014). Consequently, Black women often assume important social roles within and outside the home, leading them to value endorse androgynous gender roles, reporting high levels of stereotypical female and male characteristics (Buckley & Carter, 2005; Dade & Sloan, 2000). These experiences have shaped how Black women socialize their Black girls, emphasizing qualities such as self‐sufficiency, assertiveness and strength, qualities not similarly valued by white women (Oshin & Milan, 2019). This greater flexibility in gender roles has been linked to higher self‐esteem and more positive body image in Black girls (Buckley & Carter, 2005; Molloy & Herzberger, 2002), but likely bring them into more conflict with pre‐K‐12 teachers, who are overwhelmingly white women. Together, this small body of literature suggests Black girls are being socialized in ways that may bring psychological benefits but heightens their risks in culturally invalidating school contexts. Although there is less research on the school‐specific gendered racial socialization of Black boys, when surveyed, Black boys have demonstrated understandings of their identity that exist outside of rigid gender norms and that consider themselves capable of academic excellence (Andrews, 2009; Buckley, 2018).

#### Encounters with law enforcement

Another key context for heightened vulnerability for Black boys and Black girls is in their encounters with law enforcement officers who are part of the justice system in their schools and neighborhoods. In recent years, police officers’ use of excessive force and the horrific murders of unarmed Black people by the police have been increasingly documented in the media, leading to widespread protests for police reform. However, media coverage of police brutality have disproportionately focused on Black boys and men, rendering the victimization and murder of Black girls and women invisible because of their dual racial and gender marginalization (i.e., “intersectional invisibility”; Coles & Pasek, 2020; Purdie‐Vaughns & Eibach, 2008). Research suggests that excessive force and sexual violence are the two most common forms of reported police misconduct (Cato Institute, 2010; DeVylder et al., 2017). Further, there is evidence that Black girls are subject to lethal and excessive force from law enforcement at rates that mirror the experiences of Black boys, and are disproportionately affected by police sexual violence (Edwards et al., 2019; Tromadore, 2016). Police excessive force and sexual victimization against Black adolescents are likely rooted in their common dehumanization because of racism (Owusu‐Bempah, 2017), and gender‐specific stereotypes of Black boys being dangerous and criminal and Black girls being sexually lascivious and promiscuous. As such, Black boys and girls share common and unique vulnerabilities in their interactions with law enforcement in the U.S.

#### Sexual abuse‐to‐prison pipeline

Black girls are also at heightened risk for the sexual abuse‐to‐prison pipeline, which refers to the pathway of gendered violence through which girls are funneled into the juvenile justice system (Anderson & Walerych, 2019; Burson et al., 2019; Saar et al., 2016). Research indicates that a history of sexual abuse or violence is one of the strongest predictors of girls’ involvement in the juvenile justice system, with 31–81% of justice‐involved girls reporting histories of sexual abuse (Miller et al., 2012; Saar et al., 2016). Notably, Black girls’ contact with the juvenile justice system often occurs because of their behavioral and emotional reactions to trauma, such as truancy, running away, curfew violations, and substance use (Baumle, 2018; Saar et al., 2016). Thus, girls are criminalized for their own victimization, and this sexual abuse‐to‐prison pipeline disproportionately affects Black girls who represent the fastest growing population of incarcerated youth (Crenshaw et al., 2015; Quinn et al., 2021). Further, Black girls in the juvenile justice system often fail to receive mental health services to address their trauma symptoms and instead are subject to conditions and practices that can be retraumatizing, such as strip searches and inappropriate use of restraints (Saar et al., 2016). Following their re‐entry back into the community, these girls now face greater barriers to educational attainment and employment and are at increased risk for continued sexual victimization, continuing the vicious cycle of abuse and incarceration. This line of work underscores the importance of adopting an intersectional perspective when attempting to understand the mechanisms by which Black boys and girls are controlled, punished, and placed on a pathway that increases risk for incarceration.

#### Sex trafficking of minors

Black girls are at increased risk of sexual exploitation through modern‐day sex trafficking. More than half of U.S. human trafficking cases involve the commercial sexual exploitation of children (CSEC), or the buying, selling, or trading of sexual services from anyone under the age of 18 (Hornor, 2015; Kotrla, 2010). Approximately 200,000 children in the U.S. are victims of CSEC each year (Office of Juvenile Justice and Delinquency Prevention, 2014), with the vast majority being underage girls (Polaris, 2019; U.S. Department of Justice, 2011). Black girls constitute a disproportionate number of sexually exploited children in the U.S. (Phillips, 2015). Indeed, a third of sex trafficking victims are Black and 95% of youth identify as female (Office of Juvenile Justice and Deliquency Prevention, 2014). Although sex trafficking affects youth of all demographics, child welfare and juvenile justice involvement, economic hardship (e.g., homelessness), detachment from education, and histories of trauma, increase risk of being trafficked (Polaris, 2019). Compared to girls of other races, Black girls are more likely to experience these risk factors due to centuries of structural racism in the U.S. that have limited Black families’ access to quality housing, education, healthcare, and employment, leaving Black girls especially vulnerable to trafficking and sexual exploitation.

While Black girls are disproportionately affected by child sex trafficking, they are not recognized as victims and are punished and not protected by U.S. law enforcement. Research shows that Black youth represent ~51% of juvenile arrests for commercial sexual acts (FBI, 2019), and this percentage is significantly larger in major metropolitan areas, such as New York City, Los Angeles, Miami, and Dallas. For example, although Black girls only make up 3% of the population in Los Angeles County, they accounted for 92% of youth arrested for prostitution in this region in 2010 (Sabaté, 2012). Notably, antitrafficking efforts are often grounded in the argument that children lack the physical, mental, and emotional maturity to consent to sexual acts. However, racialized, and gendered constructions of Black girls as older, more mature, and more sexually promiscuous then their white counterparts (Epstein et al., 2017), have likely been used to deny Black girls’ access to antitrafficking protections and to justify arresting, prosecuting, and incarcerating them for prostitution (Ocen, 2015). Further, by criminalizing Black girls for their exploitation, the judicial system reinforces stereotypes about Black sexuality and deviancy, which ultimately supports the people and systems that continue to sexually exploit them. In a study conducted by the Urban Institute, sex traffickers admitted during interviews that although trafficking white women and girls would be more profitable, they believed that the penalty for trafficking Black girls would be less severe if they were caught (Dank et al., 2014). Thus, the current legal response to Black girls vulnerabilities to sex trafficking puts them at heightened risk of being targeted by sex traffickers who are keenly aware that the legal system offers little to no protections to Black girls.

##### Gendered racial socialization to cope with law enforcement encounters and sex abuse

To help youth cope with potential encounters with law enforcement and sexual abuse, parents likely engage in tailored racial socialization messages for their youth. The research on gendered socialization suggests Black boys receive more messages concerning racial barriers (i.e., preparation for bias) compared to Black girls (Peck et al., 2014; Thomas & Speight, 1999). Black girls, in contrast, tend to receive more messages seeking to instill cultural pride (Caughy et al., 2011; Peck et al., 2014). These differences may be rooted in Black parents concerns about how racial discrimination may affect their Black boys compared to their Black girls (Varner & Mandara, 2013). For example, during a series of in‐depth qualitative interviews following the murder of 17‐year‐old Trayvon Martin, parents reported that relative to Black girls, Black boys are confronted with more negative stereotypes; especially, stereotypes that portray them as dangerous (Thomas & Blackmon, 2015). Consequently, parents expressed more concern regarding the physical safety of their sons, which led them to provide specific messages on how to navigate hostile interactions with police and other experiences of racial profiling (Thomas & Blackmon, 2015). Similar messages regarding the physical safety of Back girls were absent. It is possible that Black parents’ heightened concerns for the safety of Black sons relative to their daughters will, in part, be due to gender disparities in media coverage of police violence that shapes Black parents’ concerns for their daughters (Malone Gonzalez, 2022; Samuels et al., 2021).

Moreover, scholars have recently expressed concerns that Black girls are not being prepared to manage experiences of gendered racism, including police sexual violence (Malone Gonzalez, 2019, 2022). Malone Gonzalez (2022) argues that in the same way parents provide Black boys with strategies for avoiding police physical violence, such as keeping their hands in sight, they should also provide Black girls with strategies for mitigating risk of police sexual violence. She notes, “I find strategies for black girls in the predatory talk for police sexual violence can involve explicitly ignoring officers’ commands, including continuing to drive, reaching for a phone, and calling parents during an encounter,” especially when alone or at night (Malone Gonzalez, 2022, p. 36).

## INTERVENTION AND POLICY IMPLICATIONS

Highly publicized acts of police brutality and the disproportionate effect of the COVID‐19 pandemic on Black families have led to increased concerns regarding the wide‐ranging effects of racial discrimination on the psychological adjustment and outcomes of Black youth. These concerns have been coupled by greater awareness of the unique need and responsibility to reduce risk in Black youth through interventions and policy reform. In considering the importance of history in framing the ACEs of Black boys and girls, it is key to leverage interventions that have been developed for these populations but are often overlooked by researchers and clinicians. For example, Emotional Emancipation Circles were developed by the Community Healing Network in collaboration with the Association of Black Psychologists and have been used to address historical and racial trauma among Black people, including Black youth (Barlow, 2018; Grills et al., 2016). Another advancement in this area has included capitalizing on the buffering effects of racial socialization by directly targeting and strengthening Black parents’ racial socialization competencies. This approach has shown significant promise as an effective strategy for counteracting the pervasive and enduring effects of racial discrimination on Black youth (Anderson et al., 2018; Metzger et al., 2021). However, as efforts to address racial trauma in Black youth continue, it is critical to adopt an intersectional framework that is responsive to the unique experiences and needs of different subgroups of youth. For example, it is possible that racial socialization interventions could be strengthened by attending the intersecting racial and gender identities of Black youth and equipping them the tools for coping with gendered racism.

Research on how racial discrimination varies along gendered lines also has important implications for education reform. Efforts to address racial disparities in discipline and academic outcomes are often based on the experiences of Black boys, implicitly suggesting that the experiences and needs of Black girls are the same as their male counterparts. However, studies reviewed in this article suggest that different factors contribute to the disproportionate use of exclusionary disciplinary practices against Black boys and girls. This research challenges the use of gender‐neutral approaches to educational reform and underscores the need for policies that reflect the racialized and gendered context in which exclusionary punishment is enforced.

Similarly, although police violence against Black girls has received increased public awareness with the launch of the #SayHerName campaign in 2015, efforts to understand the frequency and impact of police violence on Black girls have been challenged by the woefully inadequate amount of data on police violence (Jacobs, 2017). It is often through social media and video‐recorded footage that the public becomes aware of these incidents, and media coverage often focuses on the killings of Black boys and men, while stories of police violence against Black girls and women are buried (Jacobs, 2017; Ritchie, 2017). Further, discussions of police reform often center on excessive use of physical force, but fail to acknowledge sexual violence by police officers, which disproportionately affects Black girls. Thus, to better understand the prevalence of these incidents and identify subgroups of youth at greatest risk of victimization, it is imperative that we continue to demand greater transparency from law enforcement. Specifically, police agencies should be required to document and make publicly available the names of officers accused or convicted of sexual abuse and the details of such incidents, including the age, gender, and race of the victim(s).

While there are important gender differences in Black youths’ experiences of racial discrimination, a recurring theme across studies is the ways in which, Black boys and girls have been dehumanized and robbed the protections of childhood and adolescence that have been afforded to other youth. For youth who are repeatedly told in direct and implicit ways that their lives and pain do not matter, therapeutic approaches that focus on youth psychopathology may be experienced as further invalidating. Thus, when working with Black youth who have experienced racial trauma, it is important for clinicians to remember the numerous ways in which these youth have demonstrated extraordinary resilience and to encourage them to draw on their cultural and family strengths in their healing process.

## FUTURE DIRECTIONS AND CONCLUSION

While the current review focused on understanding Black youths’ experiences of racism along gendered lines, it is critical to also consider other aspects of their identity, such as sexual orientation, socioeconomic status, and country of origin. Relatedly, youth vary in the extent to which they ascribe importance and meaning to being Black and in the extent to which they identify as being masculine or feminine. These individual differences in racial and gender identity may affect how youth interpret and cope with experiences of gendered racism and should be considered in future research.

In sum, Black adolescents’ mental health is framed by several layers of gendered racism that affect their interpersonal experiences, their socialization experiences, and their institutional opportunities. In effect, many of these conceptions of gendered racism serve to erase the youth of Black boys and girls, through historical myths of Black male violence and dangerousness or Black female lasciviousness. Even these conceptions do not capture the full depth and nuance of how Black boys and girls experience ACEs; however, this level of analysis can serve to further reveal how Black boys and girls make sense of their adversities, and thus help to optimize treatments for and research on these populations.
